# *De novo* coiled-coil peptides as scaffolds for disrupting protein–protein interactions†
†Electronic supplementary information (ESI) available. See DOI: 10.1039/c8sc02643b


**DOI:** 10.1039/c8sc02643b

**Published:** 2018-08-07

**Authors:** Jordan M. Fletcher, Katherine A. Horner, Gail J. Bartlett, Guto G. Rhys, Andrew J. Wilson, Derek N. Woolfson

**Affiliations:** a School of Chemistry , University of Bristol , Cantock's Close , Bristol BS8 1TS , UK . Email: A.J.Wilson@leeds.ac.uk ; Email: D.N.Woolfson@bristol.ac.uk; b School of Chemistry , University of Leeds , Woodhouse Lane , Leeds LS2 9JT , UK; c Astbury Centre for Structural Molecular Biology , University of Leeds , Woodhouse Lane , Leeds , LS2 9JT , UK; d School of Biochemistry , University of Bristol , Medical Sciences Building, University Walk , Bristol BS8 1TD , UK; e BrisSynBio , University of Bristol , Life Sciences Building, Tyndall Avenue , Bristol , BS8 1TQ , UK

## Abstract

Protein–protein interactions (PPIs) play pivotal roles in the majority of biological processes. Therefore, improved approaches to target and disrupt PPIs would provide tools for chemical biology and leads for therapeutic development. PPIs with α-helical components are appealing targets given that the secondary structure is well understood and can be mimicked or stabilised to render small-molecule and constrained-peptide-based inhibitors. Here we present a strategy to target α-helix-mediated PPIs that exploits *de novo* coiled-coil assemblies and test this using the MCL-1/NOXA-B PPI. First, computational alanine scanning is used to identify key α-helical residues from NOXA-B that contribute to the interface. Next, these residues are grafted onto the exposed surfaces of *de novo* designed homodimeric or heterodimeric coiled-coil peptides. The resulting synthetic peptides selectively inhibit a cognate MCL-1/BID complex in the mid-nM range. Furthermore, the heterodimeric system affords control as inhibition occurs only when both the grafted peptide and its designed partner are present. This establishes proof of concept for exploiting peptides stabilised in *de novo* coiled coils as inhibitors of PPIs. This dependence on supramolecular assembly introduces new possibilities for regulation and control.

## Abstract 2

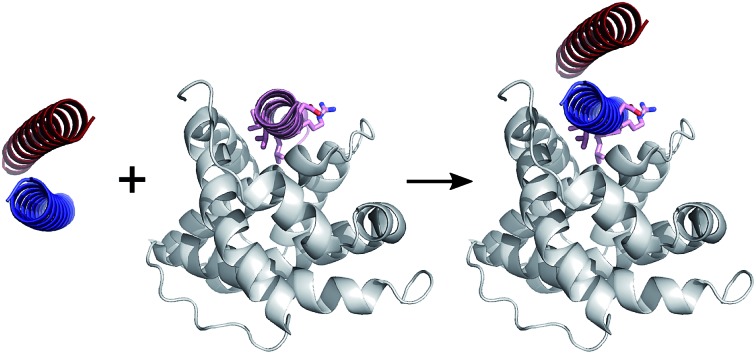
Homo- and hetero-dimeric coiled coils as scaffolds for the presentation of α-helical protein-binding motifs.

## Introduction

In healthy cells, proteins tend not to function as single entities but rather as components of dynamic, multi-component complexes. Thus, the function of a protein does not merely relate to its underlying primary and tertiary structures but also to the protein–protein interactions (PPIs) that it makes. In turn, PPIs regulate the vast majority of biological processes, which they do with exquisite control.^1^,^2^ For example, the roles of Myc, Mad and related oncogenes in transcriptional control depends on them making heterodimeric coiled-coil-based complexes with Max.^3^ Likewise in disease, mutations to or changes in expression levels of such components can lead to aberrant PPIs. Therefore, efforts to modulate PPIs have received increasing attention within the chemical-biology and drug-discovery communities;^4^–^6^ although our rudimentary understanding of signalling pathways means that it is not always clear what proteins to target to guarantee desired responses.^7^

The use of synthetic molecules to modulate PPIs is attractive for multiple reasons, including: in principle, they are orthogonal to the natural components being targeted; they can be used to facilitate temporal and titratable control over protein function to illuminate biological processes in healthy and diseased states; and chemical probes in particular can serve as springboards for subsequent drug discovery and development.^8^,^9^ That said, the design or engineering of genuinely competitive synthetic inhibitors of a PPI requires projection of recognition moieties and functional groups into what are often relatively flat, large protein surfaces. As a result, progress in this area has proven challenging.^4^–^6^ Similarly, the ability to emulate and exploit nature's regulatory mechanisms using synthetic molecules—*e.g.*, to rewire signalling processes,^10^ control localisation^11^ or initiate degradation^12^—is still in its infancy.

α-Helix-mediated PPIs have emerged as a class that is amenable to small-molecule inhibition.^13^–^15^ Considerable work in this area has led to the elaboration of several generic modalities for inhibition,^16^ including the use of mini^17^ and designed proteins,^18^–^20^ stapled peptides^21^–^27^ and foldamers,^28^,^29^ all of which mimic the topology of the helix; the development of proteomimetics^30^–^32^ that mimic the topography of a helix; and protein grafting.^33^,^34^ Such constrained peptides, which exploit the functionally optimised specificity and selectivity of natural peptide motifs, are attractive from a recognition perspective as pre-organising such motifs in a recognition competent conformation may enhance affinity;^35^ and the constraint may bring benefits such as improved stability to proteolysis^36^ and cellular uptake,^37^ which can be limiting with linear-peptide-based drugs.

Advances in methods to constrain peptides in helical conformations have led to considerable success in targeting diverse PPIs^21^,^23^ and progress towards clinical applications.^35^ Despite these advances, most approaches deviate from natural, proteinogenic amino acids invariably resulting in increased chemical complexity and, in turn, increasing the difficulty and cost of synthesis. Moreover, the constraining moiety itself potentially introduces non-canonical binding modes^38^ and off-target toxicity (*e.g.*, through cell-membrane interactions).^39^ In addition, with only a few exceptions—*e.g.*, photoresponsive^40^ and reversible^41^ constraints—current methods are not amenable to introducing regulatory control mechanisms such as those present in biological systems, *i.e.*, conformational switching.

To address some of these issues, we reasoned that α-helical coiled coils might provide suitable scaffolds for presenting motifs to disrupt PPIs. In the first aspect of our concept—which involves grafting a motif onto the outer, solvent-exposed face of a *de novo* homodimeric coiled coil—two peptide helices effectively template and stabilise each other. In the second—which uses a *de novo* heterodimeric coiled coil with only one of the peptides decorated with the motif—an element of control is introduced, as the grafted peptide is only stabilised and competent to interfere with the PPI with its partner present. Others have reported a similar system based on the natural homodimeric leucine zipper, GCN4, from yeast.^34^ Here we expand on this theme, employing *de novo* designed coiled coils over which we have considerable control in directing oligomer state, partner specificity and stability through rational peptide design.^42^,^43^

**Fig. 1:**
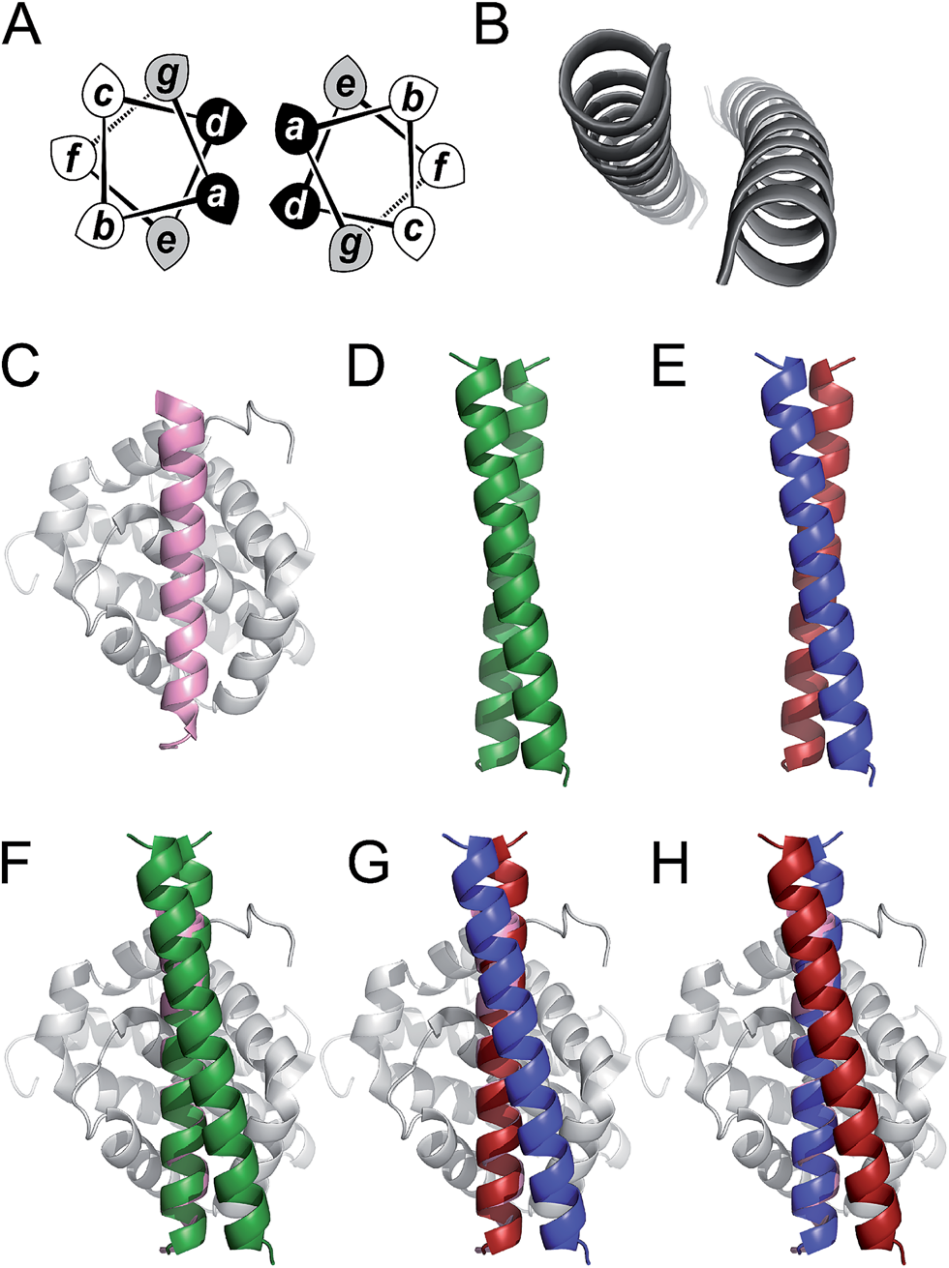
Coiled coils, MCL-1 structures and design rationale for the current study. (A) Helical-wheel diagram depicting a parallel dimeric coiled coil. Hydrophobic residues at positions a and d are shaded black and flanking residues at e and g are shaded grey. (B) The archetypal parallel, homodimeric coiled coil GCN4 (ref. 50) (PDB code: ; 2ZTA). (C) Solution structure of the MCL-1 : NOXA-B complex^63^ (PDB: ; 2JM6). The NOXA-B ligand is coloured pink, and the MCL-1 protein shaded grey. (D) Crystal structure of the *de novo* designed homodimeric coiled coil, CC-Di^42^ (PDB: ; 4DZM). (E) Schematic depicting the structure of a coiled-coil heterodimer composed of an acidic strand (red) and a basic strand (blue). (F–H) Representations of proposed MCL-1 : coiled coil assemblies generated by aligning backbone atoms of one helix from the coiled coil with those of the NOXA-B peptide. Alignments and images were generated using PyMOL [; http://pymol.org/2/].

Coiled coils are one of the most abundant, extensively studied, and well understood of all protein folds.^44^,^45^ Their sequences often have patterns of hydrophobic (H) residues spaced alternately three and four residues apart with intervening polar (P) residues to give characteristic 7-residue, or heptad repeats, HPPHPPP, usually denoted abcdefg. When configured into an α helix, this places the H residues at a and d positions resulting in a seam that drives helical association (Fig. 1). As proposed by Crick,^46^ the structural characteristic of coiled coils is “knob-into-holes” packing in which residues at a and d on one helix dock into diamond-shaped constellations formed by residues at d_–1_, g_–1_, a & d and a, d, e & a_+1_ of a neighbouring helix. Side chains at e and g flank this hydrophobic core and are frequently complementary charged residues leading to inter-helical salt bridges. The remaining residues at the b, c, and f positions are usually not part of the helix interface and offer scope to be decorated with residues of choice. This understanding of coiled-coil assembly has led to sequence-to-structure relationships and computational methods to facilitate the reliable *de novo* design of a wide variety of coiled coils. These include homo-dimers through heptamers,^42^,^47^–^50^ heteromeric complexes,^43^,^51^–^55^ and parallel or antiparallel topologies.^56^,^57^ Herein, we use parallel homodimeric (Fig. 1D) and heterodimeric (Fig. 1E) coiled coils as scaffolds for presenting binding residues.

To test our hypothesis that coiled coils could be used to scaffold motifs to inhibit PPIs, we sought a model interaction in which a relatively long and straight helical ligand binds its cognate protein along a deep groove. Of the clinically relevant helix-mediated PPIs,^13^,^58^ we chose the MCL-1/NOXA-B interaction (Fig. 1C). MCL-1 is an anti-apoptotic member of the BCL-2 family of PPIs that collectively determine cellular response to pro and anti-apoptotic stimuli.^59^ The canonical BCL-2 interaction involves docking of the BH3 domain from a family member in a helical conformation to a cleft on a multi-BH-domain partner.^60^ MCL-1 is a particularly attractive target because of its role in multiple cancers and its immunity to inhibition by other BCL-2 family inhibitors.^61^ As a result, chemical probes have been developed to validate it as a target for preclinical studies.^62^

Here we target the MCL-1/NOXA-B interaction to examine if α-helical ligands could be redesigned to be stable, potent inhibitors of PPIs without recourse to using non-proteinogenic amino acids and specialist synthetic methodologies. First, we perform a computational alanine scan to identify residues present in NOXA-B critical for MCL-1 binding. Next, we graft these residues onto the outer face (b, c, and f sites) of the *de novo* coiled-coil homodimer, CC-Di (Fig. 1F), and on each peptide of the obligate heterodimer, CC-Di-AB (Fig. 1G and H).^43^ The decorated coiled coils are characterised by solution-phase biophysical techniques, and MCL-1 binding is determined by fluorescence anisotropy competition assays. In this way, we show that inhibition requires stabilisation of the helical conformation of the synthetic ligand, and that it depends on coiled-coil formation in a manner that mirrors regulatory control observed in cellular processes. Crucially, the grafted ligands retain the selectivity profile of NOXA-B and do not inhibit interactions of the related BCL-2 family member, BCL-x_L_, and an unrelated helix mediated PPI, hDM2/p53.

## Results and discussion

### Designing coiled-coil dimers to mimic NOXA-B

**Fig. 2:**
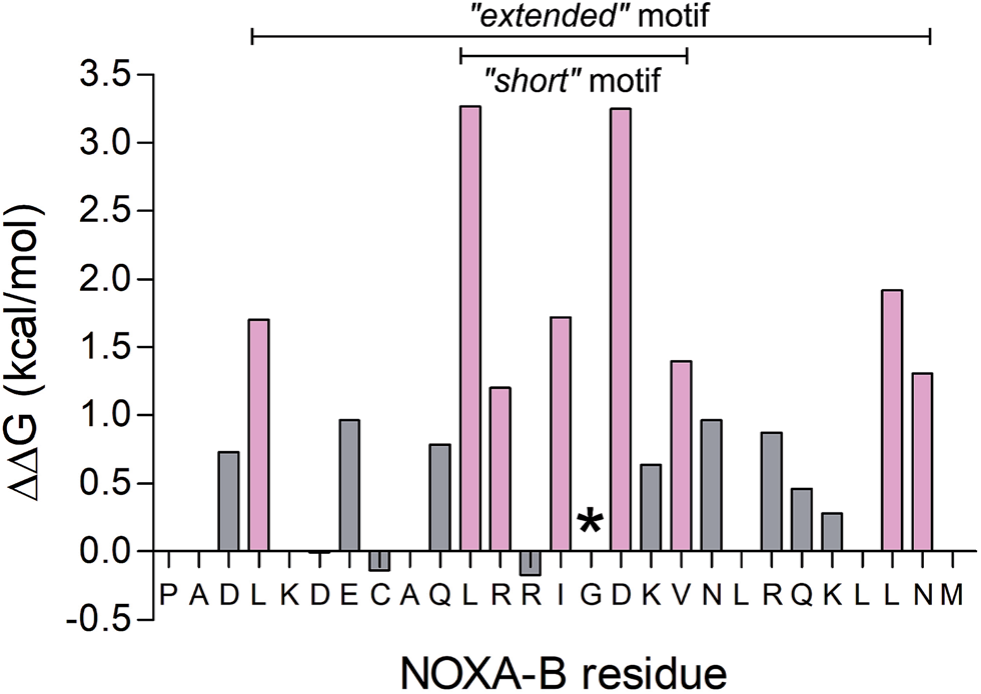
Computational alanine scan examining MCL-1/NOXA-B interaction using Robetta.^64^ Residues with a calculated ΔΔ*G* of >1 kcal mol^–1^ are coloured pink. The Gly residue, conserved across BCL-2 family members, is also highlighted (*).

To determine the most-important residues of NOXA-B for binding MCL-1, an NMR structure of MCL-1/NOXA-B (PDB ID: 2JM6) was probed by computational alanine (Ala) scan using Robetta.^59^ Briefly, this defines the interface between partners by calculating the contribution that each residue makes to the *in silico* interfacial free energy relative to an Ala mutant at that position. Arbitrarily, residues with ΔΔ*G* of >1.0 kcal mol^–1^ were considered significant contributors to binding (Fig. 2).^64^ These included a central cluster of residues—Leu11, Arg12, Ile14, Asp16, and Val18—which we refer to as the short motif (S); and Leu4, Leu25 and Asn26, which, together with S, comprise an extended motif (E1). Glycine (Gly) residues are not included in such analysis because mutation to Ala introduces bulk and may not be tolerated without changes to the backbone. Inspection of the MCL-1/NOXA-B structure revealed close packing of Gly15 against MCL-1, and the residue is highly conserved in BH3 domains across BCL-2 family members.^65^ Therefore, this residue was retained in the designs depicted in Table 1.

**Table 1:** Designed NOXA-B/coiled-coil hybrid pep-tides. Key: residues of NOXA-B identified to bind MCL-1 are underlined. These are then serially grafted onto the *de novo* coiled-coil sequences (CC-Di, green; CC-Di-A, red; CC-Di-B, blue) as indicated in underlined bold

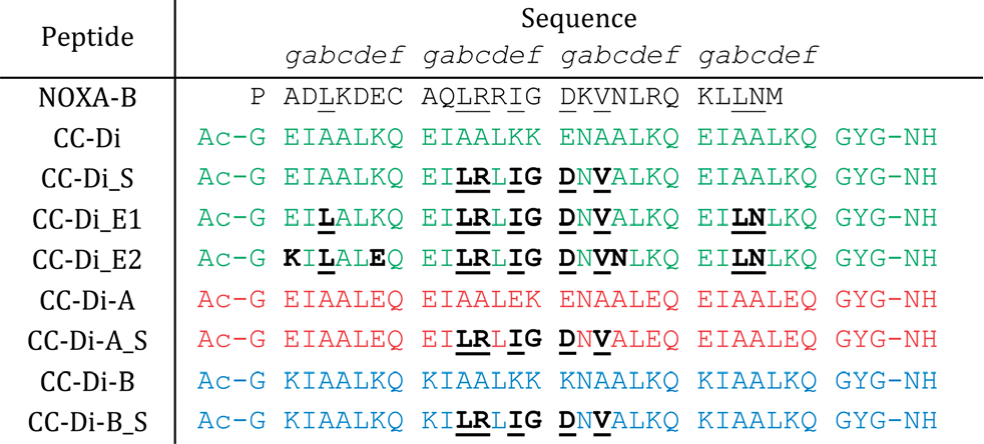

The *de novo* designed coiled-coil peptide CC-Di (Fig. 1B; PDB: ; 4DZM)^42^ was chosen as the scaffold for iterative functional design for the following reasons: first, CC-Di has been extensively characterised;^42^ second, the *de novo* design centres on the g, a, d and e sites of the heptad repeat, leaving residues at b, c and f free for functionalisation; third, related to the previous point, altering the salt-bridge patterning at g and e allows this homodimeric coiled coil to be converted to an obligate heterodimer comprising peptides CC-Di-A and CC-Di-B (Fig. 1E);^43^ fourth, it does not suffer from the oligomer state promiscuity observed for other commonly used coiled coils such as GCN4-p1;^66^ and finally, the X-ray crystal structure of CC-Di (PDB: ; 4DZM)^42^ reveals a high superhelical pitch of 226 Å, which renders the helices relatively straight (Fig. 1D). For the design process, we grafted the identified binding residues together with the conserved Gly from NOXA-B onto the sequence of CC-Di to give a series of hybrid designs (Table 1). The sequences of the NOXA-B peptide and CC-Di were aligned manually such that the binding residues from the former overlaid predominantly the b, c and f positions of the latter. This process furnished several peptides: CC-Di_S, designed to form a homodimeric coiled coil with the short motif presented on both of its outer surfaces; CC-Di_E1, to present two copies of the extended motif; and CC-Di_E2, to present the extended motifs plus two additional residues, Glu7 and Asn19, from NOXA-B that each scored ΔΔ*G* values of 0.96 kcal mol^–1^ in the computational alanine scan (Fig. 2). N.B., to incorporate Glu7, we switched the second residue to Lys to maintain the intermolecular salt bridge in the first heptad of this coiled coil. To add control over helix formation and scaffold assembly, and to reduce the number of configured binding sites to one per assembly, we also designed two heterodimeric systems: CC-Di-A_S, which is an acidic peptide containing the short motif, designed to partner peptide CC-Di-B; and CC-Di-B_S, a basic peptide with the same graft that should partner peptide CC-Di-A (Table 1).

The designed peptides were synthesised by microwave-assisted Fmoc solid-phase techniques, purified by RP-HPLC, and confirmed by mass spectrometry (ESI Fig. S1 and S3†).

### The redesigned peptides fold as α-helical dimers in solution

**Fig. 3:**
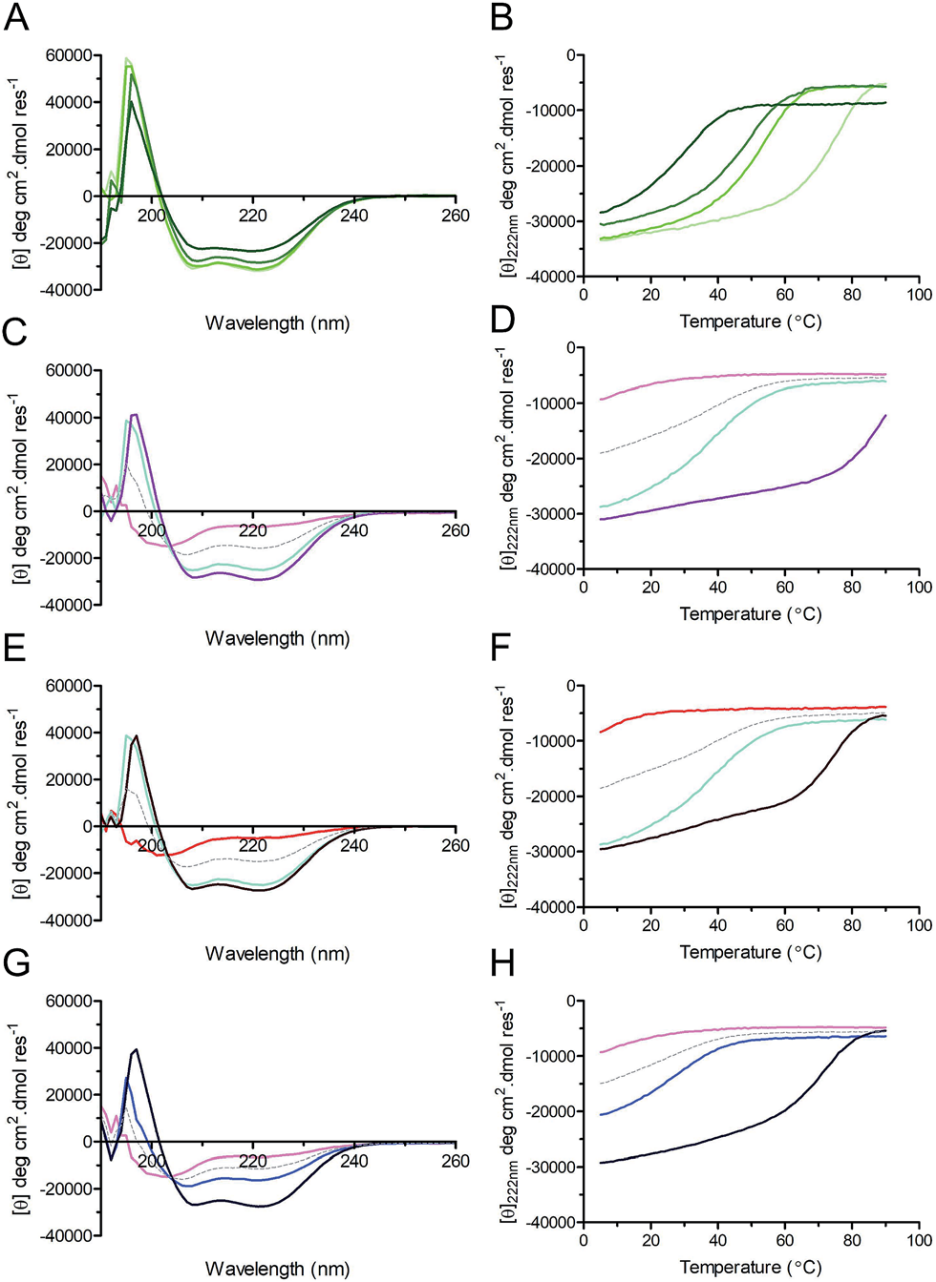
CD spectra (left) and thermal unfolding curves (right) for coiled coils, and coiled coil–NOXA-B hybrid peptides. (A & B) CC-Di (pale green), CC-Di_S (lime), CC-Di_E1 (green), CC-Di_E2 (olive). (C & D) CC-Di-A (pink), CC-Di-B (cyan), CC-Di-A : CC-Di-B mixture (purple). (E & F) CC-Di-A_S (red), CC-Di-B (cyan), CC-Di-A_S : CC-Di-B mixture (brown). (G & H) CC-Di-A (pink), CC-Di-B_S (blue), CC-Di-A : CC-Di-B_S mixture (indigo). For heteromeric assemblies (*i.e.* Panels C–H) the theoretical spectra expected should the pairs of peptides not interact are depicted by grey dashes. Conditions: all experiments were performed in phosphate-buffered saline (PBS; pH 7.4) at 50 μM concentration of each peptide. Equilibrium CD spectra were recorded at 20 °C.

Circular dichroism (CD) spectroscopy was used to examine the secondary structure and stability of designed peptides in solution (Fig. 3, Table 2). Like the parent CC-Di, at 50 μM the hybrid peptides CC-Di_S, CC-Di_E1, and CC-Di_E2 exhibited CD spectra typical of highly α-helical structures (Fig. 3A). On thermal denaturation, however, the hybrid peptides were found to be destabilised with reduced midpoints (*T*_M_) of thermal unfolding transitions relative to the parent (Fig. 3B, Table 2). This is likely a consequence of the *en bloc* changes to the sequence including the loss of a g–e salt bridge in heptad 2; and the introduction of several helix-destabilising residues,^67^ in particular the Gly residue at position 15. Indeed, across the series of homodimeric peptides prepared for this study, the *T*_M_ values consistently fell as more residue changes were made (Table 2). Regarding the heterodimeric designs, both CC-Di-A and CC-Di-A_S were largely unfolded, whereas the two CC-Di-B-based peptides showed some α helicity, but these were unstable to heating with *T*_M_ values of <40 °C (Fig. 3C–H and Table 2). However, mixing the CC-Di-A variants with the CC-Di-B-based partners (at 50 μM of each peptide) gave increased α helicities and thermal stabilities (Fig. 3C–H and Table 2); although, again, these were lower than for the parent CC-Di-A : CC-Di-B mixture. Similar results were obtained when these analyses were performed at 1 μM peptide concentration, though with expected further drops in *T*_M_ (ESI Table S5†).

**Table 2:** Summary of biophysical data

Peptide(s)	Schematic	MRE_222_^*a*^ (deg cm^2^ dmol res^–1^), (% helicity)^68^	*T*_M_ ^*b*^ (°C)	Oligomer state^*c*^	IC_50_ MCL-1/BID (μM)
CC-Di	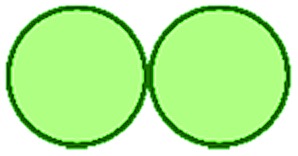	–31.650 (90%)	75	1.96	No response
CC-Di_S	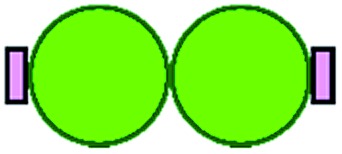	–30.930 (88%)	53	1.74	26 ± 1
CC-Di_E1	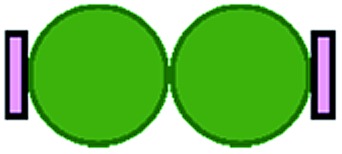	–28.259 (81%)	48	1.93	0.7 ± 0.05
CC-Di_E2	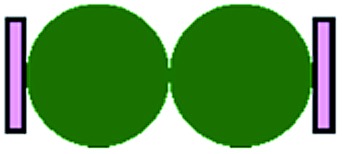	–23.091 (66%)	30	1–2^*d*^	0.09 ± 0.005
CC-Di-A	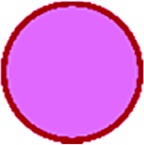	–6577 (19%)	NA (<0)	NA	No response
CC-Di-A_S	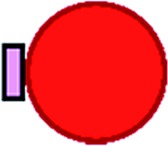	–4910 (14%)	NA (<0)	NA	21 ± 0.4
CC-Di-B	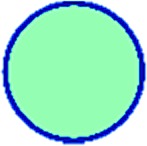	–25.088 (71%)	38	ND	No response
CC-Di-B_S	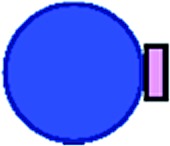	–16.247 (46%)	27	ND	No response
CC-Di-A + CC-Di-B	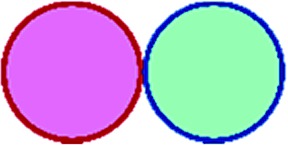	–29.154 (82%)	91	ND	Not tested
CC-Di-A_S + CC-Di-B	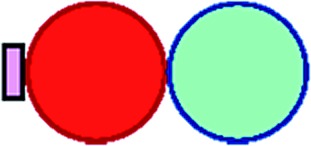	–27.395 (78%)	74	1.83	44 ± 1
CC-Di-A + CC-Di-B_S	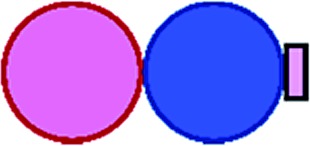	–24.490 (69%)	71	1.9	40 ± 2

^*a*^Mean residue ellipticity values at 222 nm from circular dichroism (CD) spectra recorded at 20 °C in PBS at 50 μM of each peptide.

^*b*^Midpoints of thermal denaturation curves determined by monitoring the MRE_222_ between 5 and 90 °C ramped at 40 °C h^–1^.

^*c*^Oligomeric states determined by analytical ultracentrifugation and expressed relative to monomer molecular mass.

^*d*^Data for CC-Di_E2 were fitted to monomer–dimer equilibrium.

We used sedimentation-equilibrium analytical ultracentrifugation (AUC) to determine if the grafting of NOXA-B residues into the *de novo* coiled coils, which are designed to be dimeric,^42^,^43^ affected their oligomeric state (Table 2, Fig. S6†). The AUC data for the CC-Di_S and CC-Di_E1 peptides and the hetero-combinations CC-Di-A : CC-Di-B_S and CC-Di-A_S : CC-Di-B all fitted to single ideal species and returned weights consistent with dimeric assemblies (Table 2). Data recorded at 20 °C for the most extensively decorated peptide, CC-Di_E2, fitted to monomer–dimer equilibrium returning a *K*_D_ of 7.26 μM. This is consistent with results obtained from thermal denaturation experiments monitored by CD (*T*_M_ = 16 °C at 1 μM peptide concentration; ESI Table S5†).

In summary, the biophysical analyses confirm that upon grafting NOXA-B-derived MCL-1-binding residues onto the *de novo* designed coiled-coil sequences the peptides remain folded dimers in solution at pH 7.4 and at least over the 1–50 μM range of peptide concentrations.

### The decorated coiled-coil peptides displace a reporter peptide from MCL-1

Fluorescence anisotropy (FA) competition assays were performed to define the potential inhibitory behaviour of the coiled coil–NOXA-B hybrids towards MCL-1. In these experiments, the decorated peptides—either individually for the CC-Di variants, or paired for the CC-Di-A/B systems—were titrated against MCL-1 preincubated with a fluorescently labelled tracer peptide, FITC-BID, which is derived from the wild-type BID BH3 domain (Table S3†).^24^

**Fig. 4:**
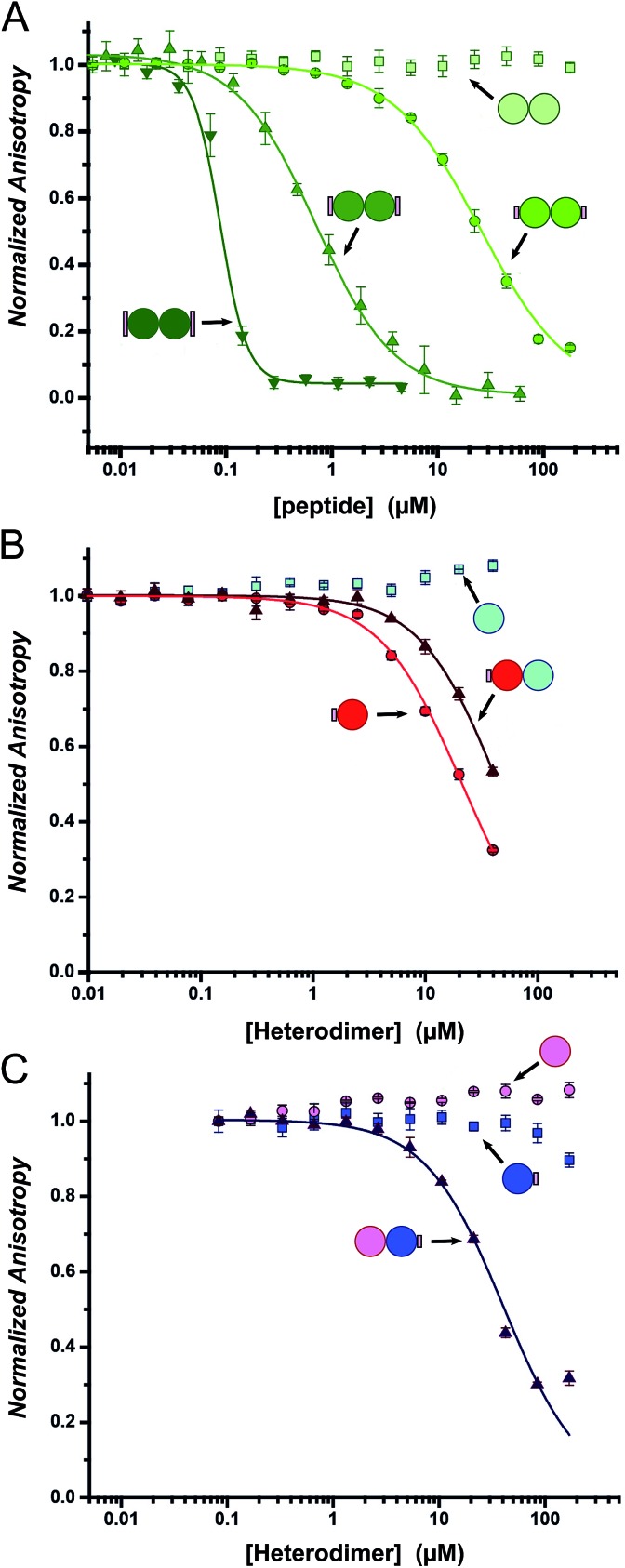
Competitive-displacement assays of hybrid peptides inhibiting the MCL-1/FITC BID interaction. (A) Titrations with the homodimeric CC-Di variants (CC-Di (pale green squares), CC-Di_S (lime circles), CC-Di_E1 (green upwards triangle), CC-Di_E2 (olive downwards triangle)); (B) the heterodimer system with CC-Di-A decorated (CC-Di-A_S (red circles), CC-Di-B (cyan squares), CC-Di-A_S : CC-Di-B mixture (brown triangles)); and (C) the heterodimer system with CC-Di-B decorated (CC-Di-A (pink circles), CC-Di-B_S (blue squares), CC-Di-A : CC-Di-B_S mixture (indigo triangles)). Conditions: 150 nM MCL-1, 25 nM FITC-BID, 21 °C, Tris buffer (50 mM Tris, 150 mM NaCl, pH 7.4) + 0.01% Triton-X-100.

As a control, the unmodified homodimeric coiled coil, CC-Di, did not displace FITC-BID (Fig. 4A). By contrast, responses were observed for all three decorated homodimeric coiled coils indicating a specific binding event conferred by the NOXA-B-binding residues grafted onto the peptides. Moreover, the half-maximal inhibitory concentrations (IC_50_) decreased significantly across the series CC-Di_S, CC-Di_E1, CC-Di_E2, indicating that the recognition surface of NOXA-B was being increasingly mimicked. Indeed, incorporation of the larger NOXA-B interface gave an inhibitory potency of ≈90 nM. For comparison, a truncated wild-type NOXA-B competitor peptide yielded an IC_50_ of 375 ± 22 nM (ESI Fig. S8b†). This demonstrates the potential power of the grafting strategy described herein to deliver MCL-1 ligands that are comparable or superior to BH3 domains. Crucially, the results imply that binding and stabilisation of CC-Di_E2 to MCL-1 are co-operative; *i.e.*, MCL-1 binding stabilises the coiled-coil assembly.

### Heterodimeric coiled coils provide control over binding

To gauge the relative importance of induced and pre-organised α helicity, we extended the competition binding assays to the designed heterodimers. We compared the binding of the decorated peptides CC-Di-A_S and CC-Di-B_S alone and when dimerised with their cognate undecorated partners CC-Di-B and CC-Di-A, respectively (Fig. 4B and C).

First, and surprisingly, peptide CC-Di-A_S bound to MCL-1 appreciably with an IC_50_ of ≈20 μM (Fig. 4B, Table 2). Moreover, this binding was impaired two-fold in the presence of CC-Di-B. We offer the following explanation for this: first, and in contrast to the other peptides of this study, CC-Di-A and CC-Di-A_S are expected to be highly anionic at neutral pH (charge ≈–7). This could augment binding through electrostatic interactions as the solvent-exposed surface of MCL-1 is somewhat basic around the NOXA-B binding groove (Fig. S7†). Thus, there might be some electrostatic steering between the highly acidic CC-Di-A_S and the MCL-1 target.^69^ In turn, this might explain the reduced binding for the CC-Di-A_S : CC-Di-B heterodimer as the highly basic CC-Di-B (anticipated charge at neutral pH ≈ +9) will effectively neutralise or screen the anionic character of CC-Di-A_S. Nonetheless, second, we can attribute some of the binding of the isolated CC-Di-A_S peptide to the grafted NOXA-B residues as unmodified CC-Di-A does not bind MCL-1 (Fig. 4C). Indeed, CC-Di-A_S possesses more MCL-1 binding residues than its nominally similarly decorated counterparts CC-Di_S and CC-Di-B_S. Specifically, and as part of the CC-Di-A background design, CC-Di-A_S has glutamic acid (Glu) at residue 7 (Table 1). This residue aligns with a Glu residue of extended binding motif from NOXA-B (Fig. 2) being noted as a possible contributor to binding in the computational alanine scan (ΔΔ*G* = 0.96 kcal mol^–1^). This highlights the multi-factorial nature of PPIs and that attention must be paid to all such factors in inhibitor design.

By contrast, when NOXA-B residues were grafted onto the CC-Di-B component of the heterodimer the behaviour was as designed: neither of the free peptides, CC-Di-B or CC-Di-B_S, competed with FITC-BID for binding to MCL-1 (Fig. 4B and C); however, when complemented by CC-Di-A, CC-Di-B_S bound with IC_50_ comparable to the CC-Di_S homodimer (Fig. 4C, Table 2). Thus, in this case non-covalent interaction with CC-Di-A biases CC-Di-B_S in favour of an α-helical and bioactive conformation, in effect entropically pre-organising it to enhance target binding affinity.

### The design constructs bind and inhibit MCL-1 selectively

**Fig. 5:**
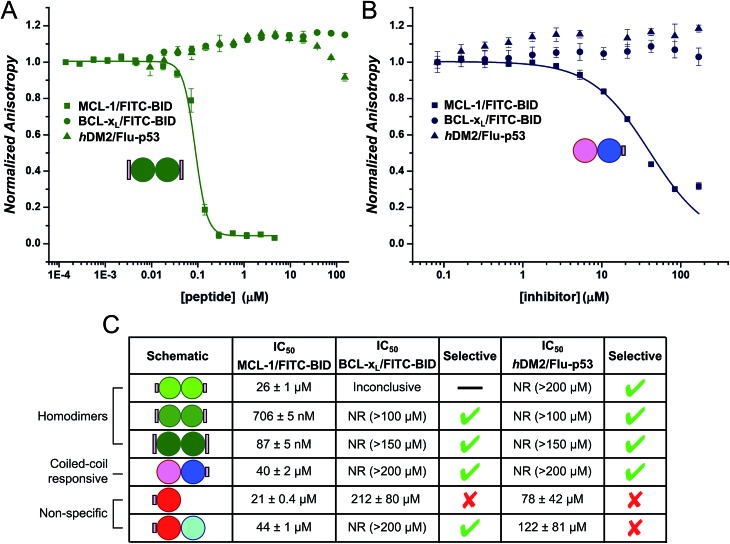
Selectivity assays of coiled-coil constructs against MCL-1/FITC-BID (squares), BCL-x_L_/FITC-BID (circles), and hDM2/Flu-p53 (triangles). With CC-Di_E2 (A) and CC-Di-B_S + CC-Di-A (B). (C) Tabulated results for all selectivity displacement assays. Conditions: 150 nM BCL-x_L_, 25 nM FITC-BID, and 150 nM hDM2, 25 nM Flu-p53.

Finally, we tested the extent to which the *de novo* coiled coil–NOXA-B hybrids that bound MCL-1 did so selectivity (Fig. 5). To do this, we employed two related protein targets, BCL-x_L_ and hDM2.^15^,^70^–^72^ Like MCL-1, BCL-x_L_ is a multi-domain anti-apoptotic BCL-2 family member. MCL-1 and BCL-x_L_ engage in both selective and promiscuous interactions with pro-apoptotic modulators and executioners within the BCL-2 family. However, unlike MCL-1, BCL-x_L_ does not recognise NOXA-B, whilst both proteins recognise BID and other BH3 sequences.^73^,^74^ The different selectivity preferences of BCL-x_L_ and MCL-1, their close relationship within the BCL-2 family, and the fact that both could be used in the competitive fluorescence anisotropy experiments using FITC-BID, made BCL-x_L_ a particularly attractive and stringent test of selectivity. hDM2 is a negative regulator of the transcription factor p53,^70^,^71^ and was also included as an interesting test of selectivity as p53 has been shown to interact with both MCL-1 and BCL-x_L_ through a transcription-independent mechanism.^75^

Data (not shown) from the titration of the homodimeric CC-Di_S against BCL-xL/FITC-BID were inconclusive, and no clear displacement/binding curve was apparent. We attribute this to aggregation of the homodimer with other assay components, possibly with the labelled reporter peptide, at higher concentrations. However, titrating CC-Di_E1 and CC-Di_E2 against either BCL-x_L_/FITC-BID or hDM2/Flu-p53 gave no observable inhibitory response (Fig. 5A, C and S8e & g†). Notably, CC-Di_E2 was titrated to a maximum concentration of 150 μM, demonstrating at least 2000-fold selectivity towards MCL-1 over BCL-x_L_ and hDM2. Similarly, the heterodimer combination that displaced FITC-BID from MCL-1, CC-Di-B_S + CC-Di-A, did not inhibit either BCL-x_L_ or hDM2 (Fig. 5B).

CC-Di-A_S and the CC-Di-A_S : CC-Di-B combination both inhibited hDM2/Flu-p53, and the former showed weak binding with BCL-x_L_/FITC-BID (Fig. 5C and S8e & g†). This reveals poor selectively by the decorated CC-Di-A_S peptide whether alone or if complexed CC-Di-B. Together with the response noted above in the assay with MCL-1/FITC-BID, this indicates further that CC-Di-A_S binds non-specifically to different protein targets. In both cases, the binding of unfolded CC-Di-A_S was stronger than with the heterodimer combination. As noted for MCL-1, the binding sites of BCL-x_L_ and hDM2 are also slightly basic.

## Conclusion

Taken together, these data demonstrate the utility of *de novo* designed coiled coils as scaffolds for PPI inhibitors. By grafting residues from the helical motif of NOXA-B found at the natural protein–protein interface onto homo and heterodimeric *de novo* coiled coils, selective inhibition of MCL-1/FITC-BID has been achieved over BCL-x_L_/FITC-BID and hDM2/Flu-p53. The inhibitory potency depends on the extent of grafting; *i.e.*, introducing more hotspot residues gives greater potency. Moreover, as demonstrated through a heterodimeric coiled-coil system, inhibition requires the presence of both partners demonstrating supramolecular control over folding, presentation, and binding by the grafted motif.

In a study by others,^34^ p53 hotspot residues have been grafted onto the natural GCN4 leucine zipper and shown to inhibit the hDM2/p53 interaction, including, upon addition of cell-penetrating functionality to the grafted peptide, in cellular models.^34^ Using such a naturally derived coiled coil can carry risks. For instance, GCN4 variants show a variety of coiled-coil oligomer states.^66^ The *de novo* designed systems introduced herein have several distinct advantages over naturally derived coiled coils: (i) they are structurally well-defined dimeric coiled-coil templates; (ii) they can be configured as a homo or heterodimer, thus allowing control over the stoichiometry of peptide : protein assemblies; (iii) the potency of inhibition can be readily controlled by the extent of grafting; (iv) coiled-coil formation is required for inhibition, *i.e.*, one helix acts as a non-covalent staple to promote helicity of the second helix bearing the PPI motif; and (v) inhibition of the target PPI is selective.

There are potential pitfalls with *de novo* systems, of course. For example, here we note promiscuous, non-selective binding to targets by the decorated variants of the acid peptide, CC-Di-A. We attribute this to a combination of the peptides harbouring a latent binding site plus having some electrostatic complementarity to regions around the target sites. However, as we illustrate by decorating the basic peptide, with *de novo* peptides this is can be remedied by redesign.

Our study establishes that *de novo* designed peptides from a synthetic-biology toolkit^42^,^43^ can be augmented with natural motifs to modulate PPIs through supramolecular assembly. We envisage that this capability might be harnessed in other ways. For example, to direct target proteins to form new or alternative quaternary interactions mediated by coiled coils decorated with two or more different binding motifs. This could be used to inhibit two or more PPIs simultaneously, or to bring together target proteins to act together or on each another. This will be the focus of our future studies.

## Experimental

### Computational alanine scan

The first model in the NMR structure of MCL-1/NOXA-B (PDB: 2JM6) was used to carry out a computational alanine scan on the Robetta^64^ alanine scanning server (; http://www.robetta.org). Contributions of >1 kcal mol^–1^ to the binding interface were recorded as significant.

### Synthesis of coiled-coil peptides

Rink amide Chem-Matrix resin was purchased from PCAS Biomatrix Inc. (St-Jean-sur-Richelieu, Canada). Fmoc-l-amino acids and dimethylformamide (DMF) were obtained from AGTC Bioproducts (Hessle, UK); all other reagents were obtained from Sigma-Aldrich (Gillingham, UK). Peptides were prepared by standard Fmoc solid-phase techniques on a Liberty Blue microwave peptide synthesiser (CEM; Mathews, NC, U.S.A.) using repeated steps of amino acid coupling and Fmoc deprotection. Coupling was achieved by diisopropylcarbodiimide (DIC)/6-chloro-1-hydroxybenzotriazole (Cl-HOBt) activation in DMF on H-Rink Amide (ChemMatrix®) resin (0.1 mmol). Fmoc deprotection was performed by treatment with 20% morpholine in DMF. For peptides containing an aspartic acid residue, 5% formic acid was added to the deprotection solution to minimise aspartimide formation during chain elongation.^76^ Following assembly, the N-terminus of each peptide was acetylated (acetic anhydride (3 eq.), DIPEA (4.5 eq.) in DMF for 30 min). Cleavage of the peptide from the resin, and concomitant removal of sidechain protection, was achieved by treating the resin-attached peptides with a cocktail of trifluoroacetic acid (TFA)/H_2_O/triisopropylsilane (38 : 1 : 1 v/v, 10 mL) for 3 h at RT. Resin was removed by filtration before the peptide was precipitated by the addition of ice-cold diethyl ether and centrifuged. Diethyl ether was then decanted, and the peptide pellet dissolved in 1 : 1 H_2_O/MeCN, frozen and lyophilised. Peptides were purified by HPLC (using a Kromatek (semi-micro, 5 μm, 100 Å, 10 mm ID × 150 mm L) C18 reverse-phase column) employing a linear gradient (at 3 mL min^–1^) of 20% to 80% MeCN in water (each containing 0.1% TFA). Fractions thought to contain the peptide of interest were analysed by analytical reverse-phase HPLC (Phenomenex Kinetex C18 column (5 μm particle, 4.6 × 100 mm)) and MALDI-TOF mass spectrometry. Those fractions found to contain exclusively the product of interest were pooled and lyophilised. Analytical HPLC of the final product revealed a purity of >95% (Fig. S3†). Successful synthesis was confirmed by mass spectrometry (see Fig. S5 and Table S1†).

### Synthesis of monomeric native peptides and analogues

Fluorescently labelled peptide FITC-BID was prepared as previously described.^24^ Flu-p53 was purchased from Peptide Protein Research Ltd. (Bishops Waltham, UK). wt NOXA-B was prepared in house, the details of which are provided in the ESI,† with successful synthesis confirmed by ESI-MS (Fig. S2†) and HPLC (Fig. S4†). Sequences of FITC-BID, Flu-p53, and wt NOXA-B peptides are provided in Table S2.†

### Protein expression and purification

MCL-1,^24^ Bcl-x_L_,^24^ and hDM2 (ref. 77) proteins were expressed and purified as described previously. High-resolution mass-spectrometry data are provided in Fig. S5 & Table S3.†

### Circular dichroism spectroscopy

CD spectra were collected using a JASCO J810 spectropolarimeter coupled to a Peltier temperature controller. Peptide-containing solutions were freshly prepared in PBS (pH 7.4) with concentrations calculated from UV absorption at 280 nm (*ε*(Tyr) = 1280 mol^–1^ cm^–1^).^78^ Samples were examined at 50 μM concentration for lone peptides and 50 + 50 μM for two peptide heterodimeric examples, in a 1 mm quartz cuvette. Homodimeric, and heterodimeric pairs of peptides, found to be both folded and to bind MCL-1, were further examined at 1 μM in a 1 cm quartz cuvette. Thermal denaturation experiments were conducted by ramping temperature from 5 to 90 °C at 40 °C h^–1^. Full spectra (260–190 nm, 5 scans) were recorded at 5 °C and 20 °C, while the CD signal at 222 nm was recorded across the full temperature range at 1 °C intervals (1 nm interval, 1 nm bandwidth, 16 s response time). Raw data was normalised for peptide concentration & length, and cuvette pathlength to give mean residue ellipticity (MRE; deg cm^2^ dmol res^–1^).

### Analytical ultracentrifugation

Analytical ultracentrifugation (AUC) was performed at 20 °C in a Beckman Proteomelab XL-A or Beckman Proteomelab XL-I analytical ultracentrifuge using an An-60 Ti rotor and 2-channel centrepieces. Sedimentation equilibrium experiments were prepared in PBS (137 mM NaCl, 2.7 mM KCl, 8.2 mM Na_2_HPO_4_ and 1.8 mM KH_2_PO_4_) at 50 μM peptide concentration for homomeric assemblies and 50 μM peptide concentration of both peptides for heteromeric assemblies and to 120 μL. The reference channel was loaded with 130 μL of PBS solution. Equilibrium distributions were measured twice per speed, in 4 krpm increments, and with rotor speeds from 40 to 60 krpm. Data were fitted to single ideal species models using Ultrascan II (; http://www.ultrascan.uthscsa.edu). A better fit for CC-Di_E2 data was found using monomer–dimer equilibrium with a fixed monomer mass. 95% confidence limits were obtained by Monte Carlo analysis of the fits. The partial specific volume for each of the peptides and the buffer density were calculated using Ultrascan II.

### Fluorescence anisotropy

Assays were carried out in 96 or 384 well Optiplates and were scanned using a Perkin Elmer EnVision™ 2103 MultiLabel plate reader. Fluorescein-labelled peptides were examined using an excitation and emission wavelength of 480 nm and 535 nm respectively (dichroic mirror 505 nm). All assays were performed in Tris buffer: (50 mM Tris, 150 mM NaCl, pH 7.4), with additives stipulated. Direct titrations and competition assays were configured and performed using minor modifications to those described previously,^24^ and are detailed in full in the ESI.†

## Conflicts of interest

There are no conflicts to declare.

## Supplementary Material

Supplementary information
