# Supplementary material for: De novo coiled-coil peptides as scaffolds for disrupting protein–protein interactions
Source: Chem Sci. 2018 Aug 7;9(39):7656–65. doi: 10.1039/c8sc02643b (PMC6182421; doi:10.1039/c8sc02643b)
Supplement: Supplementary file 1 [file SC-009-C8SC02643B-s001.pdf]

Electronic Supplementary Material (ESI) for Chemical Science.
This journal is © The Royal Society of Chemistry 2018

Supporting Information for:

De novo coiled-coil peptides as scaffolds for disrupting protein-protein interactions

Jordan M. Fletcher,[1,†] Katherine A. Horner,[2,3,†] Gail J Bartlett,[1] Guto G. Rhys,[1] Andrew J. Wilson,[2,3,]* and Derek N. Woolfson[1,4,5]*

Author Affiliations:

[1]School of Chemistry, University of Bristol, Cantock's Close, Bristol BS8 1TS, UK

[2]School of Chemistry, University of Leeds, Woodhouse Lane, Leeds LS2 9JT, UK

[3]Astbury Center for Structural Molecular Biology, University of Leeds, Woodhouse Lane, Leeds, LS2 9JT, UK.

[4]School of Biochemistry, University of Bristol, Medical Sciences Building, University Walk, Bristol BS8 1TD, UK

[5]BrisSynBio, University of Bristol, Life Sciences Building, Tyndall Avenue, Bristol, BS8 1TQ, UK

†These authors contributed equally to this work

*Corresponding authors: A.J.Wilson@leeds.ac.uk; D.N.Woolfson@bristol.ac.uk

TABLE OF CONTENTS

1 Supplementary Methods

1.1 Synthesis of *wt* NOXA-B S3
1.2 Analytical Ultracentrifugation S3
1.3 Fluorescence Anisotropy – Direct Binding S3
1.4 Fluorescence Anisotropy – Competition Assays S4

2 Supplementary Tables

S1 Summary of MALDI-TOF mass spectrometry data for coiled-coil peptides S5
S2 Sequences of assay peptides S5
S3 Summary of HRMS results for purified proteins S5
S4 Summary of AUC data S6
S5 Summary of CD data collected at 1 μM peptide concentration S6

3 Supplementary Figures

S1 MALDI-TOF mass spectrometry results for coiled-coil peptides. S7
S2 ESI-MS results for *wt* NOXA-B S7
S3 Analytical HPLC spectra for purified coiled-coil peptides S8
S4 Analytical HPLC for *wt* NOXA-B S9
S5 HRMS data for purified proteins MCL-1, BCL-x_L, and *h*DM2 S10
S6 AUC fits and residuals for coiled-coil peptides S11
S7 Surface rendering, and electrostatic potential of MCL-1 S13
S8 Supplementary Fluorescence Anisotropy data S13

4 Supplementary References S17

1 SUPPLEMENTARY METHODS

1.1 Synthesis of *wt* NOXA-B

Fmoc-protected amino acids were purchased from Novabiochem (Merck), or Sigma-Aldrich. The peptide was prepared using a microwave-assisted automated peptide synthesiser (Liberty Blue; CEM) on Rink amide MBHA resin (0.057 mmol) using standard Oxyma/DIC chemistry with systematically repeated steps of coupling and deprotection (20% piperidine in DMF) interspaced with washing (note: The Arg6 residue was double-coupled). Once assembly was complete, the resin-attached peptide was *N*-terminally acetylated (acetic anhydride (10 eq.), DIPEA (10 eq.) in DMF for 2 h) before being washed and dried. The peptide was then cleaved from the resin with simultaneous removal of side-chain protection by treatment with a cocktail of trifluoroacetic acid/H_2O/triisopropylsilane (95:2.5:2.5 v/v, 3 × 2 mL) for 2 h at room temperature. Resin was removed by filtration, and the peptide precipitated by the addition of ice-cold Et_2O (25 mL) and centrifuged. Supernatant was removed, and the peptide pellet dried under a stream of nitrogen, before being dissolved in H_2O and freeze-dried.

Crude *wt* NOXA-B was purified by HPLC in two stages. Firstly, using a linear gradient at 12 mL/min of 5-50% acetonitrile in water (each containing 0.1% TFA) across a RediSep®Rf gold C18 reverse-phase column (Teledyne Isco); and secondly, by MD-HPLC using a Jupiter Proteo (Phenomenex) preparative column employing a linear gradient of acetonitrile in water (each containing 0.1% formic acid). Successful synthesis was confirmed by analytical HPLC and ESI-MS ($[M+3H]^{3+}$: 749.7887 (Obs.); 749.7889 (Calc.); See Fig. S2).

1.2 Analytical Ultracentrifugation

Analytical ultracentrifugation (AUC) was performed at 20 °C in a Beckman Proteomelab XL-A or Beckman Proteomelab XL-I analytical ultracentrifuge using an An-60 Ti rotor and 2-channel centrepieces. Sedimentation equilibrium experiments were made up in PBS (137 mM NaCl, 2.7 mM KCl, 8.2 mM Na_2HPO_4 and 1.8 mM KH_2PO_4) at 50 μM peptide concentration for homomeric assemblies and 50 μM peptide concentration of both peptides for heteromeric assemblies and to 120 μL. The reference channel was loaded with 130 μL of PBS solution. Equilibrium distributions were measured twice per speed, in 4 krpm increments, and with rotor speeds from 40 to 60 krpm. Data were fitted to single ideal species models using Ultrascan II (http:/www.ultrascan.uthscsa.edu). A better fit for CC-Di_E2 data was found using monomer-dimer equilibrium with a fixed monomer mass. 95% confidence limits were obtained by Monte Carlo analysis of the fits. The partial specific volume ($\bar{v}$) for each of the peptides and the buffer density were calculated using Ultrascan II.

1.3 Fluorescence Anisotropy – Direct Binding

MCL-1/FITC-BID Direct Titration
Titration of MCL-1 into FITC-BID was performed in a 96-well plate in Tris Buffer (50 mM Tris, 150 mM NaCl, pH 7.4) + 0.01% Triton-X-100 with the concentration of MCL-1 starting at 3.75 μM, diluted over 12 points in a 1/2 regime with [FITC-BID] fixed at 25 nM. Plates were read after 1 hour and 21 hours incubation. Assays were performed in triplicate (both test wells and blank control wells).

BCL-x_L/FITC-BID Direct Titration
Titration of BCL-x_L into FITC-BID was performed in a 384-well plate in Tris Buffer + 0.01% Triton-X-100 + 0.02 mg/mL BSA with the concentration of BCL-x_L starting from 5 μM, diluted over 16 points in a 1/2 regime with [FITC-BID] fixed at 25 nM. Plates were read after 1 hour and 21 hours incubation. Assays were performed in triplicate (both test wells and blank wells).

Flu-p53/hDM2
Direct titration performed as described previously.[1]

1.4 Fluorescence Anisotropy – Competition Assays

MCL-1/FITC-BID Competition Assays
MCL-1/FITC-BID competition assays were performed in Tris Buffer (50 mM, Tris, 150 mM NaCl, pH 7.4) + 0.01% Triton-X-100 with [MCL-1] fixed at 150 nM. Assays were performed in triplicate (both test wells and blank wells). Plates were read after 1-2 hours and 20-21 hours incubation, data shown is from 20-21 hours incubation.

BCL-x_L/FITC-BID Competition Assays
BCL-x_L /FITC-BID competition assays were performed in Tris Buffer (50 mM, Tris, 150 mM NaCl, pH 7.4) + 0.01% Triton-X-100 + 0.02 mg/mL BSA with [BCL-x_L] fixed at 100 nM. For each plate, a BAK sequence used previously by us, was titrated against BCL-x_L /FITC-BID as a positive control. For the positive control, both test and blank wells were run as single titrations, for all other competition assays, test wells were run in triplicate. Plates were read after 1-2 hours and 20-21 hours incubation, data shown is from 20-21 hours incubation.

Flu-p53/hDM2 Competition Assays
Flu-p53/hDM2 competition assays were performed in Tris Buffer (50 mM, Tris, 150 mM NaCl, pH 7.4) + 0.01% Triton-X-100 with [*h*DM2] fixed at 150 nM. For each plate, Nutlin-3a was titrated against Flu-p53/hDM2 as a positive control. For the positive control, both test and blank wells were run as single titrations, for all other competition assays, test wells were run in triplicate. Plates were read after 1-2 hours incubation and 20-21 hours incubation, data shown is from 1-2 hours incubation.

2 SUPPLEMENTARY TABLES

Table S1. Summary of MALDI-TOF mass spectrometry data for coiled-coil peptides prepared in the present study.

Peptide	Sequence	Monoisotopic MW (Da)	M/Z ($[M+H]^+$)
CC-Di	Ac-GEIAALKQEIAALKKENAALKQEIAALKQGYG-NH	3407.9	3407.7
CC-Di_S	Ac-GEIAALKQEILRLIGDNVALKQEIAALKQGYG-NH	3462.9	3462.8
CC-Di_E1	Ac-GEILALKQEILRLIGDNVALKQEILNLKQGYG-NH	3590.0	3589.9
CC-Di_E2	Ac-GKILALEQEILRLIGDNVNLKQEILNLKQGYG-NH	3633.0	3633.9
CC-Di-A	Ac-GEIAALEQEIAALEKENAALEQEIAALEQGYG-NH	3411.7	3411.7
CC-Di-A_S	Ac-GEIAALEQEILRLIGDNVALEQEIAALEQGYG-NH	3465.8	3465.8
CC-Di-B	Ac-GKIAALKQKIAALKKKNAALKQKIAALKQGYG-NH	3404.1	3404.1
CC-Di-B_S	Ac-GKIAALKQKILRLIGDNVALKQKIAALKQGYG-NH	3460.1	3460.8

Table S2 Sequences of assay peptides. Abbreviations used are as follows: Fluorescein isothiocyanate (FITC); 6-Aminohexanoic acid (Ahx); Norleucine (Nle); 6-Carboxyfluorescein (Flu).

Peptide	Sequence
FITC-BID	(FITC)-Ahx-EDIIRNIARHLAQVGDS[Nle]DRSIW-NH
Flu-p53	Ac-SQETFSDLWKKPENNVC^{Flu}-NH
wt NOXA-B	Ac-AAQLRRIGDKVNLRQKLLN-NH
BAK	Ac-Ahx-GQVGRQLAIIGDDINR-NH

Table S3 Summary of deconvoluted high-resolution mass spectrometry (HRMS) results for purified proteins MCL-1, BCL-x$_L$, and *h*DM2.

Protein	Expected Average Mass (Da.)	Observed Mass (Da.)
MCL-1	17,737.2	17,736.0
BCL-x$_L$	17,489.5	17,488.5
*h*DM2	12,680.5	12,679.6

Table S4 Summary of analytical ultracentrifugation (AUC) data.

Data fitted to a single ideal species.

	Average $\bar{v}$ ($cm^3.g^{-1}$)	Returned Mass	$\frac{Returned\ Mass}{Theoretical\ Dimer\ Mass}$	95% Confidence Interval
CC-Di_S	0.7618	6010	0.87	5994 - 6025
CC-Di_E1	0.7666	6912	0.96	6895 - 6936
CC-Di-A_S & CC-Di-B	0.7636	6273	0.91	6255 - 6292
CC-Di-A & CC-Di-B_S	0.7575	6678	0.97	6647 - 6709

Data fitted to a monomer-dimer equilibrium.

	Average $\bar{v}$ ($cm^3.g^{-1}$)	Returned Dissociation Constant (μM)	95% Confidence Interval
CC-Di_E2	0.7618	7.3	6.8–7.8

Table S5 Summary of CD data collected at 1 μM for lone peptides, or 1 + 1 μM for pairs of peptides. †Mean Residue Ellipticity values at 222 nm recorded at 20 ˚C. ‡ Midpoint of thermal denaturation observed by monitoring CD at 222 nm whilst ramping temperature from 5 to 90 ˚C at 40 ˚C/h.

Peptide(s)	MRE_{222} (deg cm^2.dmol res^{-1}) †	T_M (˚C) ‡
CC-Di_S1	-22,421	36
CC-Di_E1	-23,630	32
CC-Di_E2	-11,081	16
CC-Di-A_S + CC-Di-B	-27,031	57
CC-Di-A + CC-Di-B_S	-27,082	51

3 SUPPLEMENTARY FIGURES

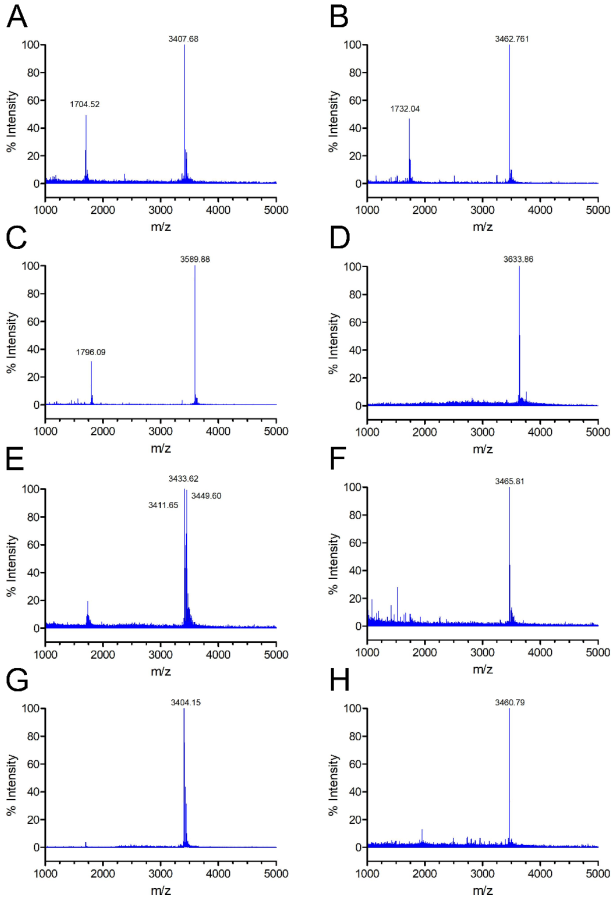

Figure S1 MALDI-TOF mass spectrometry results for coiled-coil peptides. Panel A: CC-Di; Panel B: CC-Di_S; Panel C: CC-Di_E1; Panel D: CC-Di_E2; Panel E: CC-Di-A; Panel F: CC-Di-A_S; Panel G: CC-Di-B; Panel H: CC-Di-B_S.

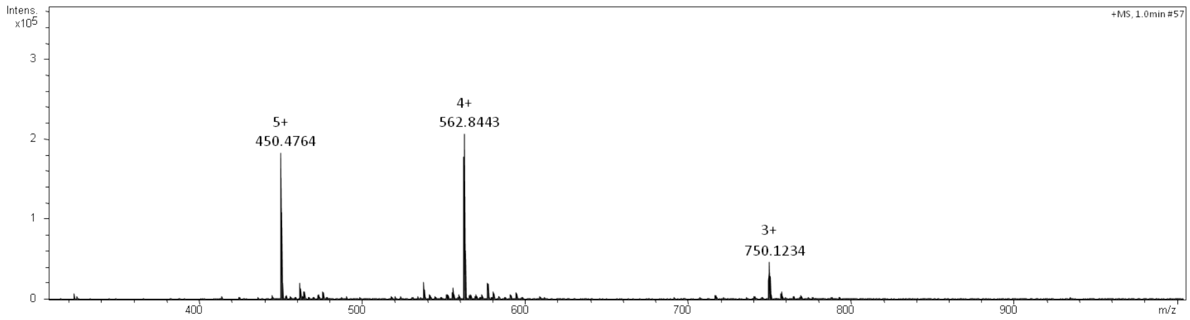

Figure S2 ESI-MS results for *wt* NOXA-B

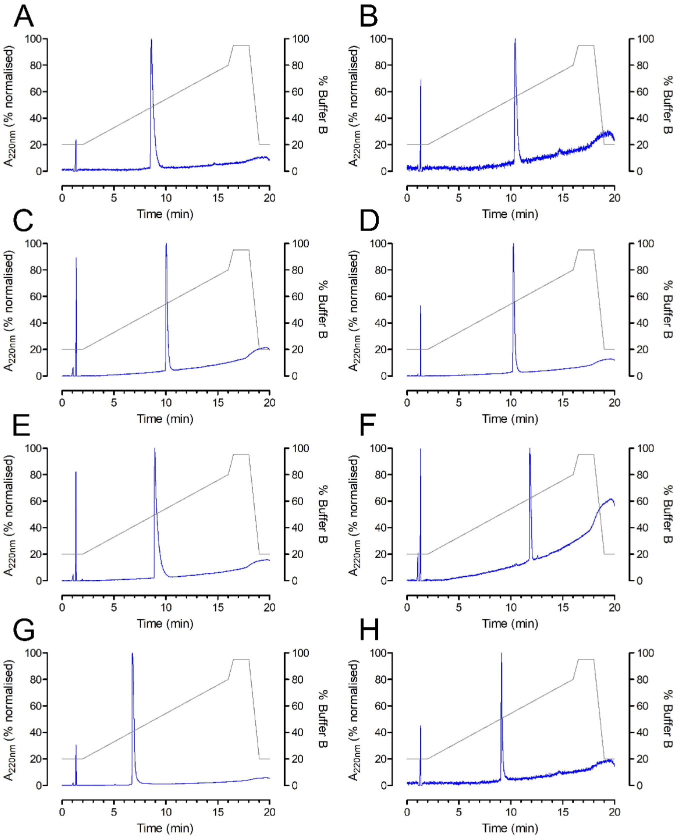

Figure S3 Analytical HPLC spectra for purified coiled-coil peptides recorded at 220 nm. HPLC was performed using a C18 reverse-phase column (Phenomenex Kinetex C18 column (5 µm particle, 4.6 × 100 mm) and a gradient of Buffers A (0.1% TFA in H_2O) and B (0.1% TFA in MeCN) as shown in grey. Panel A: CC-Di; Panel B: CC-Di_S; Panel C: CC-Di_E1; Panel D: CC-Di_E2; Panel E: CC-Di-A; Panel F: CC-Di-A_S; Panel G: CC-Di-B; Panel H: CC-Di-B_S.

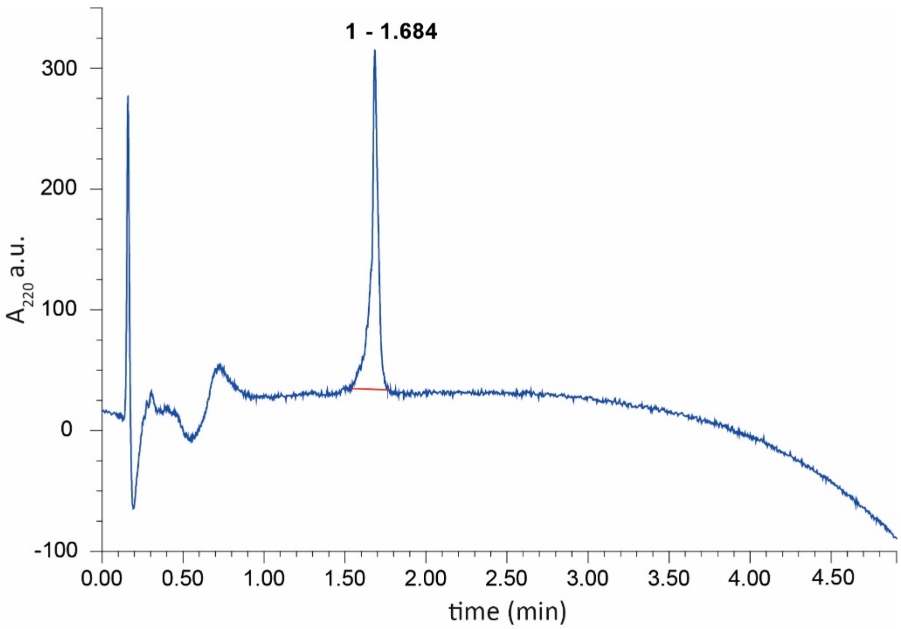

Figure S4 Analytical HPLC for *wt* NOXA-B. The peptide was eluted from the column (Acsentis express C18) by applying a linear gradient of 5-95% acetonitrile in water (both containing 0.1% TFA) over 4.9 mins.

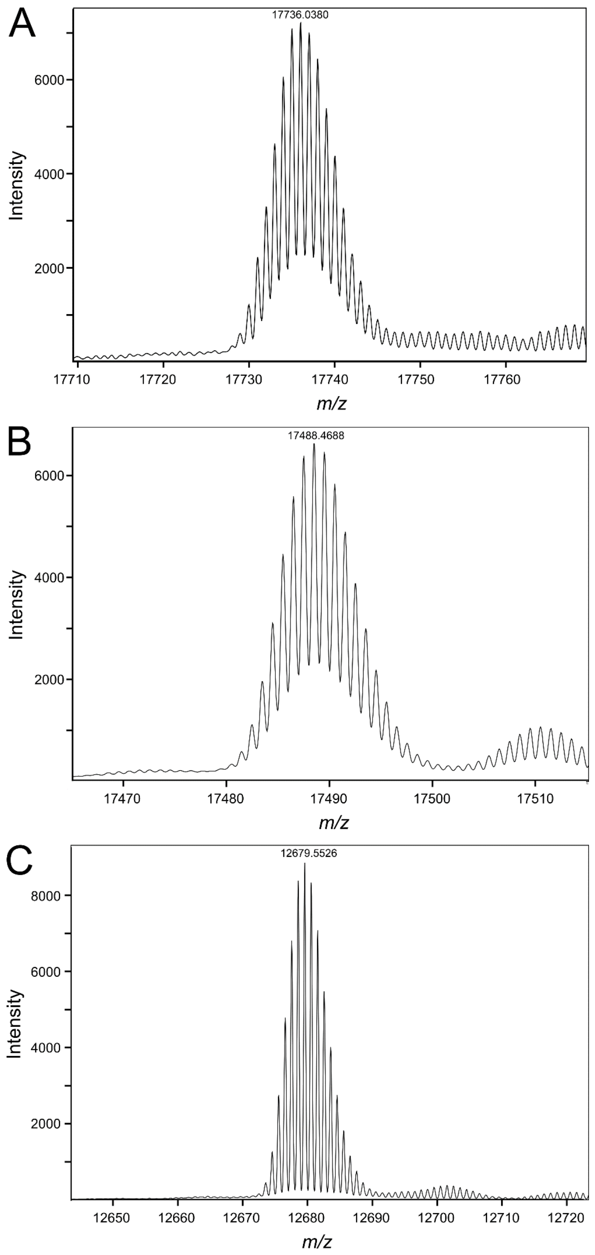

Figure S5 HRMS data for purified proteins MCL-1 (Panel A), BCL-x$_L$ (Panel B), and *h*DM2 (Panel C).

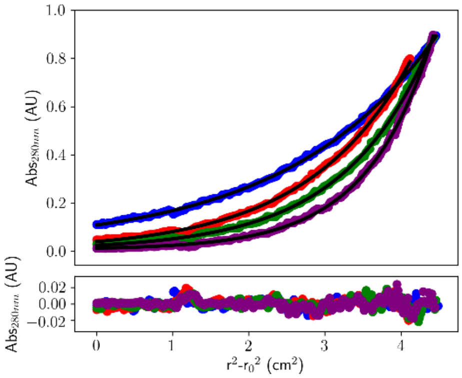

Figure S6a Analytical Ultracentrifugation fits (top) and residuals (bottom) for peptide CC-Di_S. Rotor speeds were 40,000 rpm (blue); 48,000 rpm (red); 52,000 rpm (green); 60,000 rpm (purple). The fits are shown for a single ideal species of mass 6010 Da.

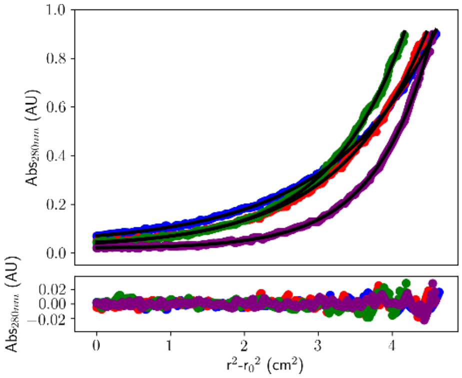

Figure S6b Analytical Ultracentrifugation fits (top) and residuals (bottom) for peptide CC-Di_E1. Rotor speeds were 44,000 rpm (blue); 48,000 rpm (red); 52,000 rpm (green); 60,000 rpm (purple). The fits are shown for a single ideal species of mass 6912 Da.

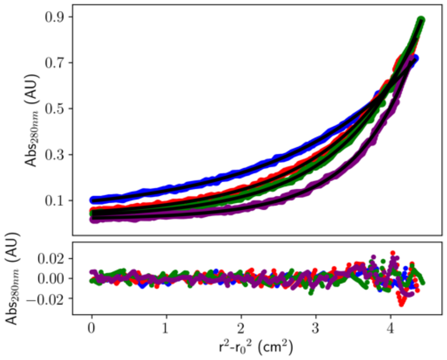

Figure S6c Analytical Ultracentrifugation fits (top) and residuals (bottom) for peptide CC-Di_E2. Rotor speeds were 44,000 rpm (blue); 48,000 rpm (red); 52,000 rpm (green); 60,000 rpm (purple). Data was fitted to a monomer-dimer equilibrium with a fixed monomer mass, returning a K_d of 7.26 μM.

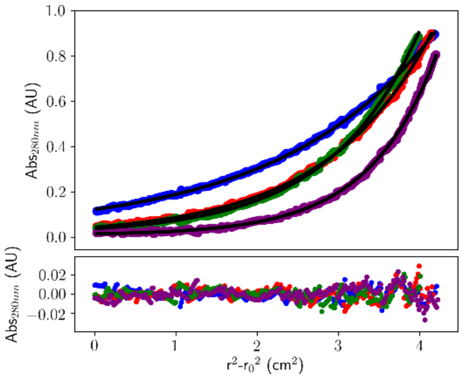

Figure S6d Analytical Ultracentrifugation fits (top) and residuals (bottom) for peptide CC-Di-A_S plus CC-Di-B. Rotor speeds were 40,000 rpm (blue); 48,000 rpm (red); 52,000 rpm (green); 60,000 rpm (purple). The fits are shown for a single ideal species of mass 6273 Da.

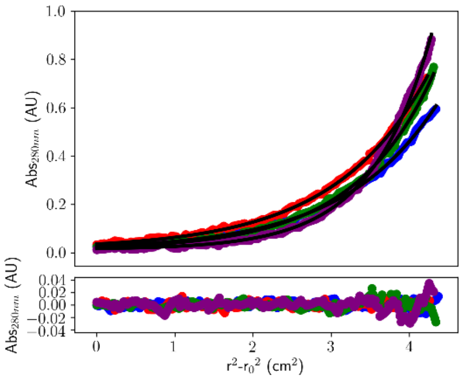

Figure S6e Analytical Ultracentrifugation fits (top) and residuals (bottom) for peptide CC-Di-A plus CC-Di-B_S. Rotor speeds were 44,000 rpm (blue); 48,000 rpm (red); 52,000 rpm (green); 60,000 rpm (purple). The fits are shown for a single ideal species of mass 6678 Da.

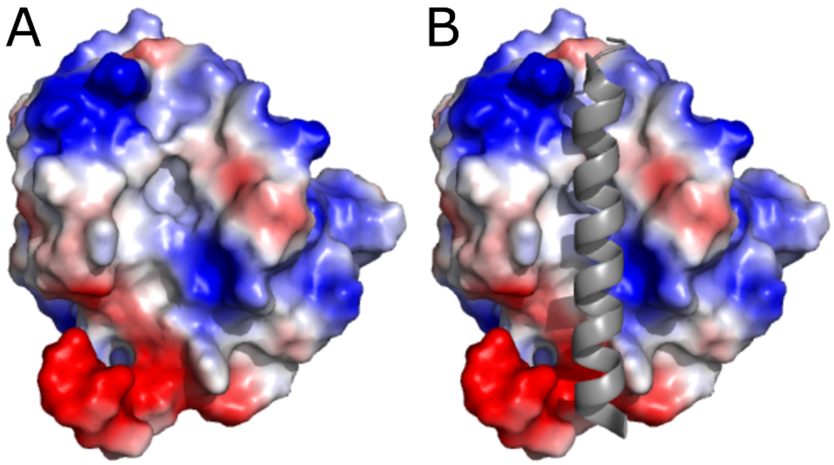

Figure S7 Surface rendering and electrostatic potential of MCL-1 depicted alone (A), and with bound NOXA-B (Grey; B) (PDB: 2JM6). Acidic regions are highlighted in red, whilst basic regions are shaded blue. Images generated using PyMOL [http://pymol.org/2/].

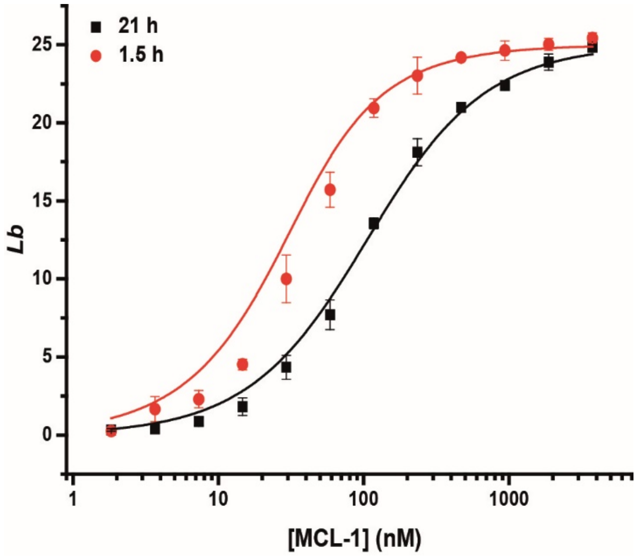

Figure S8a Direct titration of MCL-1 into FITC-BID to give an K_d = 92 ± 5 nM (21 h), which is consistent with that described previously (50 ± 20 nM).[2]

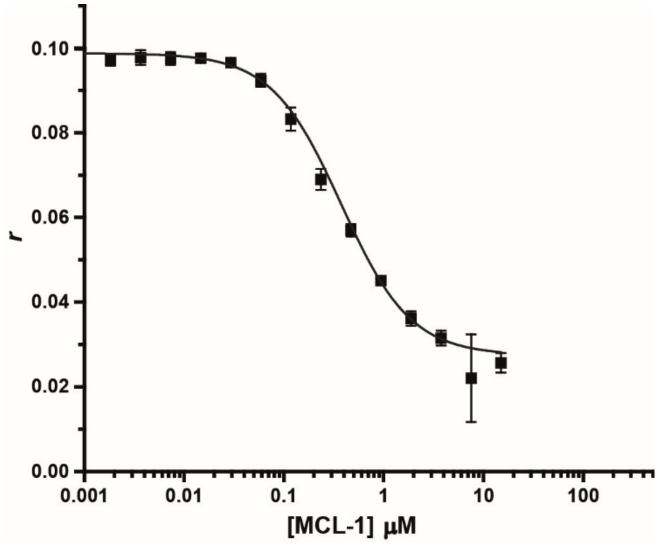

Figure S8b Representative fluorescence-anisotropy competition assay for the inhibition of MCL-1/FITC-BID interaction by a control *wt* NOXA-B peptide. IC_{50} = 375 ± 22 nM

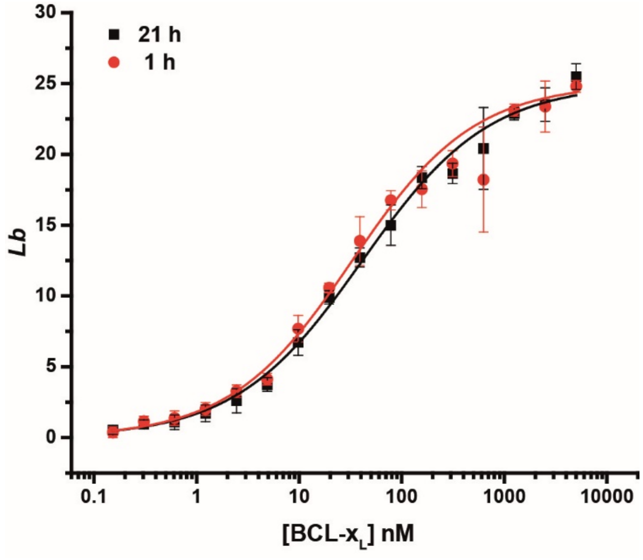

Figure S8c Direct titration of BCL-x_L into FITC-BID, data fit to a logistic model to give an EC_{50} of 41 ± 6 nM (21 h), which is consistent with that described previously (21 ± 7 nM).[2]

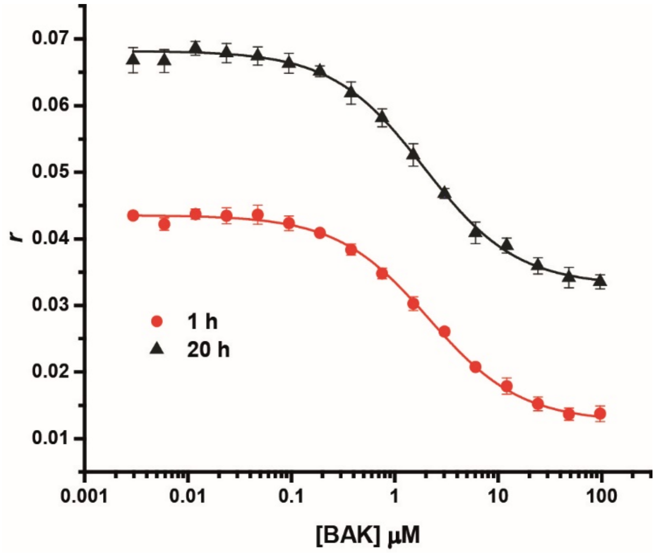

Figure S8d: Representative competitive fluorescence-anisotropy assay for the inhibition of BCL-x_L/FITC-BID by a control BAK peptide: $IC_{50} = 1.9 \pm 0.1$ µM (21 h).

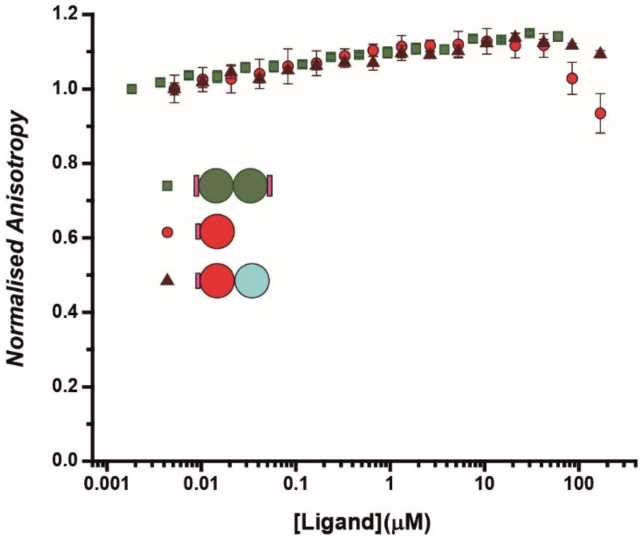

Figure S8e Competitive fluorescence-anisotropy assay for the inhibition of BCL-x_L/FITC-BID by homodimer CC-Di_E1, monomer CC-Di-A_S and heterodimer CC-Di-A_S + CC-Di-B. No observable binding response for CC-Di_E1 or CC-Di-A_S + CC-Di-B.

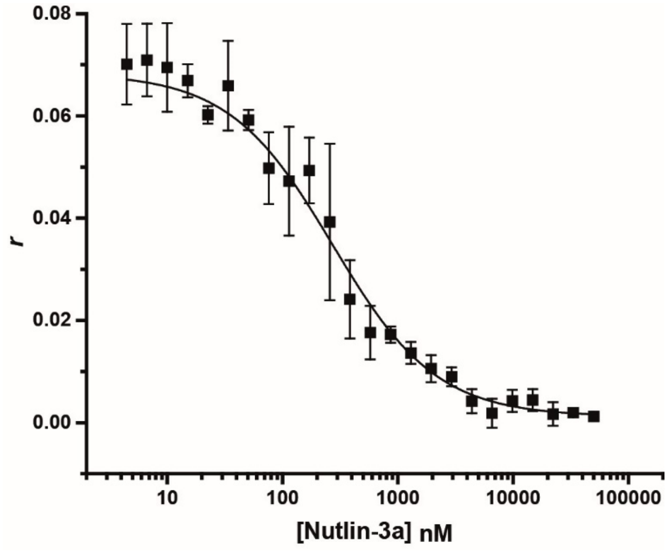

Figure S8f Representative fluorescence-anisotropy competition experiment for the inhibition of Flu-p53/*h*DM2 by Nutlin-3a control to give an IC_{50} of 273 ± 41 nM.

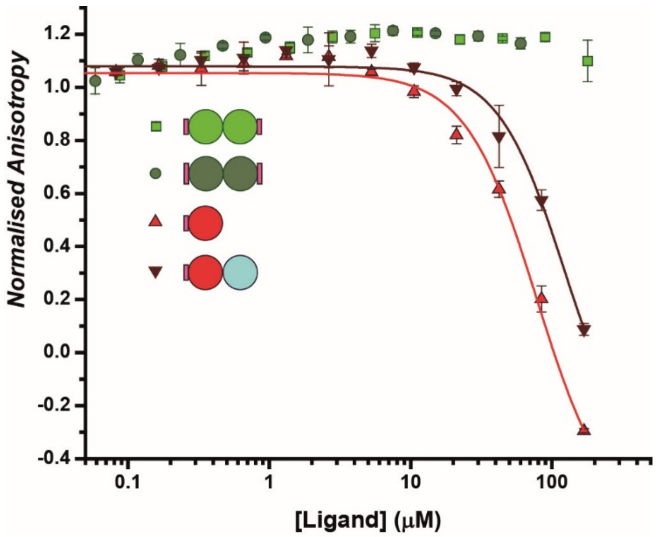

Figure S8g Fluorescence-anisotropy competition assay for the inhibition of p53/*h*DM2 by homodimers CC-Di_S and CC-Di_E1, monomer CC-Di-A_S and heterodimer CC-Di-A_S + CC-Di-B. No inhibitory effect observed for CC-Di_S and CC-Di_E1. Both CC-Di-A_S and heterodimer CC-Di-A_S + CC-Di-B inhibited the interaction with IC_{50} values of 78 ± 42 μM and 122 ± 81 μM respectively.

4 SUPPLEMENTARY REFERENCES

(1) Plante, J. P.; Burnley, T.; Malkova, B.; Webb, M. E.; Warriner, S. L.; Edwards, T. A.; Wilson, A. J., Oligobenzamide proteomimetic inhibitors of the p53-*h*DM2 protein-protein interaction. *Chem Comm.* **2009,** 5091-5093.
(2) Miles, J. A.; Yeo, D. J.; Rowell, P.; Rodriguez-Marin, S.; Pask, C. M.; Warriner, S. L.; Edwards, T. A.; Wilson, A. J., Hydrocarbon constrained peptides - understanding preorganisation and binding affinity. *Chem. Sci.* **2016,** *7*, 3694-3702.
